# A Freshwater Fish Dataset for Visual Recognition with Manually Localized ROIs and SAM-Derived Instance Masks

**DOI:** 10.1038/s41597-026-07552-1

**Published:** 2026-06-17

**Authors:** Shakib Absar, Iftekhar Ahmed, Md Istiaque Khalique, Mohammad Shorfuzzaman, Md. Mahfuzur Rahman, Asrar U. Haque, Md. Saidur Rahman Kohinoor

**Affiliations:** 1 Interdisciplinary Computer Science (InteX) Research Lab, Sylhet, 3100 Bangladesh; 2 Department of Software Engineering, College of Engineering and Advanced Computing, Alfaisal University, Riyadh, 11533 Saudi Arabia; 3 Department of Information and Computer Science, King Fahd University of Petroleum and Minerals, Dhahran, 31261 Saudi Arabia; 4 Interdisciplinary Research Center for Intelligent Secure Systems, King Fahd University of Petroleum and Minerals, Dhahran, 31261 Saudi Arabia; 5 Department of Computer Science, King Faisal University, Al Ahsa, 31982 Saudi Arabia

## Abstract

The SylFishBD dataset addresses the critical need for automated identification of local fish species in Bangladesh’s dynamic aquatic ecosystems and bustling fish markets. It comprises 9,075 high-resolution images of 9 prevalent freshwater species, captured under uncontrolled, real-world conditions over 7 months to capture seasonal variations in appearance, freshness, and market presentation. Unlike prior datasets limited to controlled studio settings, SylFishBD faithfully replicates the complexity of operational fish markets: diverse lighting (natural daylight to fluorescent), varied viewpoints (top-down, angled, close-up), cluttered backgrounds (ice, trays, water, scales), and natural occlusions (vendor hands, overlapping fish). Each image contains exactly one clearly centered fish instance, annotated with a tight bounding box and a high-precision binary segmentation mask generated using the Segment Anything Model (SAM). All images are standardized to 500  × 500 pixels, organized hierarchically by species, and accompanied by comprehensive metadata, enabling seamless integration into machine learning pipelines. The dataset supports a wide range of computer vision tasks, including classification, object detection, instance segmentation, and morphological analysis, without requiring additional preprocessing. By bridging the gap between laboratory-based datasets and authentic market environments, SylFishBD serves as a robust, publicly available benchmark for developing deployable models in real-world aquaculture, trade transparency, price monitoring, and regulatory oversight. It empowers researchers and practitioners to advance automated species recognition, freshness assessment, and fair market practices in one of the world’s most active fish-producing regions.

## Background & Summary

Bangladesh, a vital South Asian nation, boasts one of the world’s most extensive networks of rivers, wetlands, canals, and ponds, making it a global hotspot for aquatic biodiversity. Approximately 70% of its fish production comes from inland freshwater sources, with the remaining contributions from marine (sea) and brackish water systems, positioning Bangladesh as a key player in the global fisheries sector. Freshwater fish, often colloquially referred to as freshwater fish in local dialects, serve as a fundamental dietary staple for the population, providing essential proteins, vitamins, and minerals to over 170 million people. Beyond nutrition, the fisheries sector is a cornerstone of Bangladesh’s economy, generating significant revenue through domestic trade, exports (particularly of species like hilsa and shrimp), and employment. It supports an estimated 17–18 million livelihoods, including full-time fishers, part-time workers, traders, processors, and transporters, with women playing a crucial role in post-harvest activities. The sector contributes about 3–4% to the national GDP and accounts for over 60% of animal protein intake, highlighting its socio-economic importance. However, challenges such as overfishing, habitat degradation due to climate change (e.g., rising sea levels and salinity intrusion), pollution from industrial effluents, and unsustainable aquaculture practices threaten this vital resource. In comparison to marine fisheries, freshwater systems offer higher accessibility for small-scale operations but face greater vulnerability to environmental changes. Marine fisheries, focused on the Bay of Bengal, emphasize species suited for export and industrial processing, but they are prone to issues like illegal, unreported, and unregulated (IUU) fishing and cyclones. Table 1 provides a detailed comparison between freshwater and marine fish contributions in Bangladesh, illustrating their respective roles, challenges, and economic impacts.

**Table 1:** Fish Production Comparison.

Category	Freshwater Fish	Marine Fish
Contribution	~70% of total fish production	~30% of total fish production
Primary Use	Domestic consumption, local markets	Export markets, some domestic consumption
Biodiversity	High diversity (e.g., carp, catfish, tilapia)	Diverse (e.g., hilsa, shrimp, pomfret)
Economic Impact	Supports rural livelihoods, small-scale fishing	Major export revenue, industrial fishing
Challenges	Misidentification in markets, overfishing	Overexploitation, environmental degradation

**Fig. 1:**
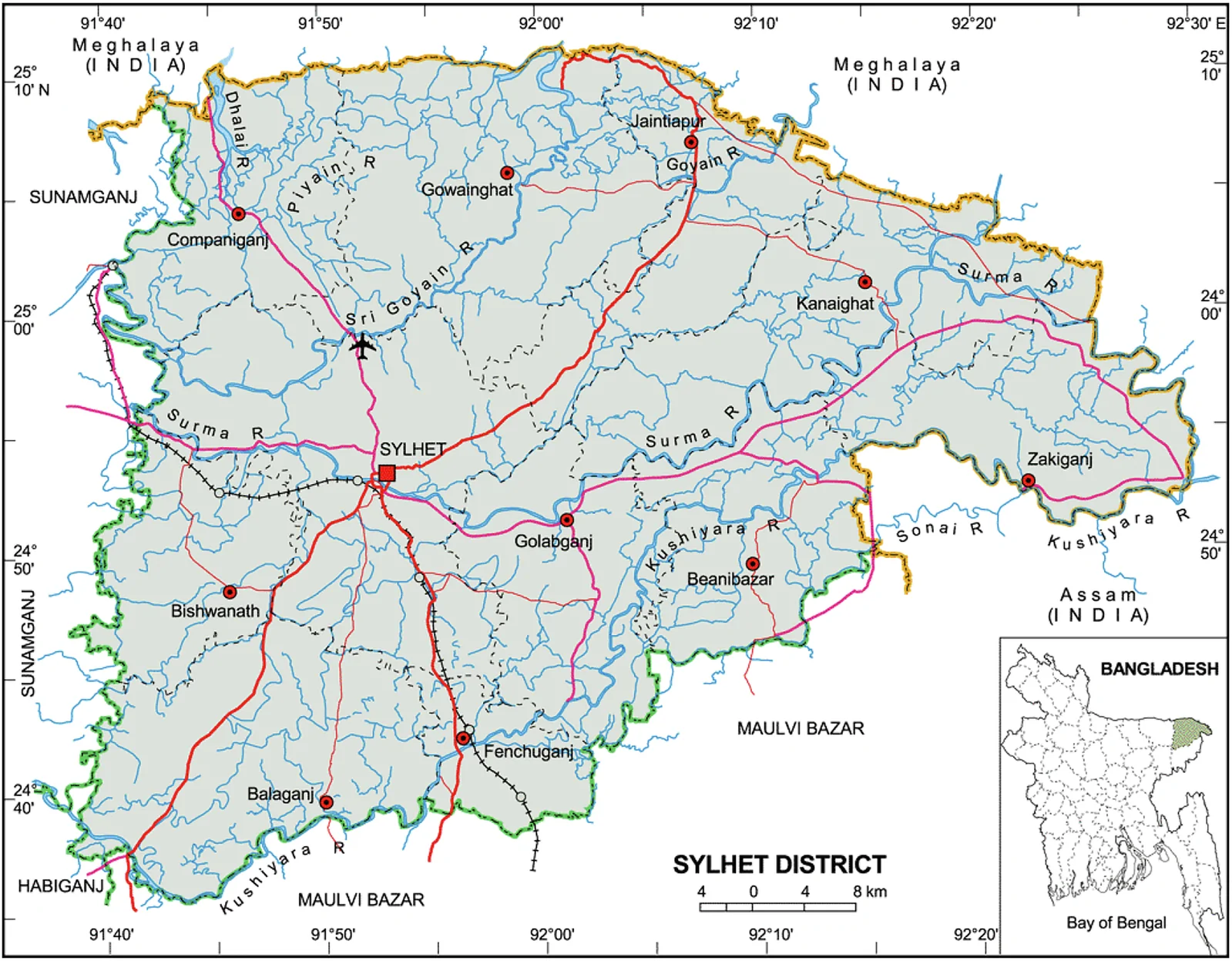
Map of Bangladesh, Highlighting Sylhet.

In Sylhet, located in northeastern Bangladesh (see Fig. 1 for a map highlighting Sylhet’s position in the southeast region of South Asia and its proximity to northern Bangladesh’s aquatic ecosystems), local fish markets are central to daily life. Vendors sell a wide variety of fish species to consumers, ranging from experienced buyers to young adults new to market shopping. However, the visual similarity among many freshwater species often leads to mislabeling, pricing errors, and confusion, particularly for those without expert knowledge. This challenge is compounded by the region’s rich biodiversity, with numerous species unique to Bangladesh’s freshwater ecosystems compared to other Asian regions.

Bangladesh’s freshwater fish species stand out in the Asian context due to the unique Bengal Delta ecology, which supports a higher number of endemic and migratory species compared to neighboring countries. While shared species exist (e.g., carps introduced via aquaculture), Bangladesh’s wetland-dominated landscape fosters greater diversity. Table 2 elaborates on this by comparing key freshwater species across Asian regions, mapping similarities and differences, and highlighting Bangladesh’s unique profile.

**Table 2:** Freshwater Fish Species in Bangladesh vs. Other Asian Regions.

Region	Key Freshwater Species	No. of Species	Unique Characteristics
Bangladesh	Rohu, Catla, Mrigal, Tilapia, Pangasius, Carp	~260	High diversity due to riverine ecosystems
India	Rohu, Catla, Grass Carp, Mahseer	~220	Similar species, less wetland diversity
Thailand	Tilapia, Snakehead, Catfish	~180	Aquaculture-dominated, fewer native species
Vietnam	Pangasius, Tilapia, Climbing Perch	~150	Focus on catfish for export
Myanmar	Carp, Catfish, Featherback	~200	River-based, less commercialized

The datasets listed provide valuable resources for addressing species identification challenges in Sylhet’s markets. Notably, the SylFishBD dataset, with 9,075 images and annotations under diverse, real-world conditions, is particularly suited for developing robust classification models to mitigate misidentification in local markets.

Existing datasets, such as BengalDeltaFish^1^, BD-freshwater-fish^2^, BanFish^3^, BDFreshFish^4^, and Fish-Pak^5^, have attempted to address fish identification but fall short due to their limitations. Most contain limited images collections or rely on controlled capture settings, such as uniform backgrounds or fixed lighting, which do not reflect the real-world complexity of market environments. In contrast, datasets from Western countries often focus on seawater fish species, which dominate their aquatic research due to their coastal ecosystems and commercial fishing industries. This leaves a gap in robust datasets for freshwater and sweet water fish, particularly in South-East Asia. The dataset DeepFish^6^ is primarily marine-focused, providing a large number of underwater images across diverse marine habitats with annotations for segmentation, classification, and size estimation, which is valuable for computer vision model benchmarking but less tailored for freshwater market environments. Additionally, the global freshwater fish trophic interactions dataset^7^ compiles extensive ecological data covering dietary and trophic relationships among numerous freshwater species worldwide, enriching ecological and biodiversity studies beyond visual identification.

The SylFishBD dataset addresses this gap with a restricted yet robust collection of 9,075 annotated images of nine common freshwater fish species, collected over 6–7 months from Sylhet’s markets. Each image, captured under varied lighting, angles, and backgrounds, includes segmentation masks, which outline the fish to support advanced computer vision tasks, such as automated species identification. This makes SylFishBD particularly valuable for South-East Asian aquatic research, where freshwater ecosystems are critical to biodiversity and food security, and it complements the seawater-focused datasets prevalent in Western research. The dataset’s real-world conditions and comprehensive annotations make it ideal for building machine learning models that can assist vendors, consumers, and researchers in accurately identifying fish species, reducing market errors, and supporting sustainable fisheries management. By providing a publicly accessible resource, SylFishBD empowers stakeholders across Bangladesh and South-East Asia to develop practical, technology-driven solutions for aquatic biodiversity challenges. Table 3 compares SylFishBD with other regional datasets, highlighting its advantages in scale, annotation depth, and real-world applicability. While SylFishBD covers nine of the most commercially significant freshwater species in Bangladesh’s Sylhet region, we acknowledge that this represents a fraction of the approximately 260 freshwater species documented in the country. The current dataset was designed as a targeted, high-quality benchmark focusing on species most frequently encountered in local markets. Future work aims to expand coverage to additional species, including less common and regionally specialized varieties, to improve the generalizability of models trained on this dataset.

**Table 3:** Gap Analysis of existing fish datasets.

Dataset	No. of Classes	No. of Images	No. of Annotations	No. of Masks	Comments
BengalDeltaFish^1^	33	4560	6037	0	Suitable for classification, limited diversity
BD-freshwater-fish^2^	12	4389	0	0	Limited images/images, controlled backgrounds
BanFish^3^	18	2506	0	0	Fixed backgrounds, unsuitable for real-world
BDFreshFish^4^	8	3233	2400	0	Controlled backgrounds, limited images/images
Fish-Pak^5^	6	915	0	0	Fixed backgrounds, very limited images/images
Global Trophic^7^	3617	N/A	Trophic data	N/A	Global ecosystem interactions, not image-based
DeepFish^6^	20+	40,000	Multiple	Multiple	Marine focus, segmentation and size estimation
**SylFishBD**	**9**	**9075**	**9075**	**9075**	**freshwater, south-eastern region, bounding-box, mask**

## Methods

The SylFishBD dataset comprises 9,075 images of nine prevalent fish species from local fish markets in Sylhet, Bangladesh, designed to advance automated fish species identification for applications in price monitoring, market transparency, and consumer education. Unlike prior datasets with limited images and controlled settings, SylFishBD captures real-world variability, including diverse backgrounds, lighting, and angles, collected over 7 months to account for seasonal changes. The dataset is organized into a Folder, with each images annotated for species classification. images are stored in JPEG format, with annotations provided in JSON files compatible with common deep learning frameworks.

### Data Collection

The SylFishBD dataset was collected from local fish markets in Sylhet, Bangladesh over 6–7 months, capturing seasonal variation and diverse real-world conditions. Images were taken using smartphones (e.g., Samsung Galaxy S21, iPhone 12) under uncontrolled lighting, angles, and backgrounds to reflect practical market scenarios. Species were identified by an experienced fish vendor and verified using standard taxonomic references. A subset of 500 images was re-evaluated by a second expert, achieving 99.4% agreement, indicating high labeling reliability.

The dataset is currently limited to Sylhet, and variations in fish appearance across other regions of Bangladesh may affect model generalizability. Future work will include data from additional regions. All images were systematically organized to ensure data integrity and support annotation in Table 4.

**Table 4:** Data description with visualization and class names.

Class Name	Description	Random Sample
Rui	Colour: Silver-coloured with slightly reddish hue Body: Streamlined Average Length: 30–50 cm Average Weight: 1–3 kg Habitat: Rivers	
Telapia	Colour: Olive grey to silvery blue Body: Laterally compressed, vertical stripes Average Length: 20–35 cm Average Weight: 0.5–1 kg Habitat: Freshwater bodies	
Mrigel	Colour: Greyish green back with silvery side Body: Streamlined and elongated Average Length: 35–60 cm Average Weight: 0.5–2 kg Habitat: Fast-flowing streams and rivers	
Katla	Colour: Greyish dorsal, whitish belly Body: Broad head, upturned mouth Average Length: 30–60 cm Average Weight: 1–3 kg Habitat: Rivers and lakes	
Boal	Colour: Silvery body Body: Elongated and compressed, broad head Average Length: 50–100 cm Average Weight: 1.5–4 kg Habitat: Large rivers and lakes	
Pabda	Colour: Silvery grey back, white belly Body: Flat and compressed Average Length: 15–25 cm Average Weight: 0.1–0.3 kg Habitat: Clear and muddy rivers, ponds, streams	
Ilish	Colour: Silver with bluish or greenish back Body: Streamlined Average Length: 40–60 cm Average Weight: 1–3 kg Habitat: Coastal water bodies and rivers	
Koi	Colour: Black/blue/white/yellow Body: Elongated Average Length: 20–40 cm Average Weight: 0.03–0.25 kg Habitat: Ponds and rivers	
Kalibaush	Colour: Silvery with slightly reddish dorsal Body: Hump on back Average Length: 40–70 cm Average Weight: 1–3 kg Habitat: Lakes and rivers	

## Camera Specifications

Camera specifications are detailed in Table 5, and the images were captured using high-resolution smartphone cameras (Samsung Galaxy S21 and iPhone 12) under uncontrolled, real-world conditions, including:

- Variable lighting: natural daylight, shaded stalls, fluorescent or LED market lights
- Diverse viewpoints: top-down, angled, side profiles, and close-ups
- Complex backgrounds: fish displayed on ice, plastic trays, metal surfaces, or stacked with other species
- Natural occlusions: vendor hands, water droplets, scales, or overlapping specimens

**Table 5:** Camera specifications.

Camera Model	Chipset/Processor	Display	Camera Specifications
Samsung Galaxy S21	Qualcomm SM8350 Snapdragon 888 (5 nm), Octa-core	Dynamic AMOLED 2X, 120Hz, 1300 nits, 6.2 inches, 1080 × 2400 pixels (421 ppi)	Triple: 12 MP, f/1.8 (wide), 12 MP, f/2.2 (ultrawide), 64 MP, f/2.0 (telephoto)
iPhone 12	Apple A14 Bionic (5 nm), Hexa-core	Super Retina XDR OLED, 625 nits, 6.1 inches, 1170 × 2532 pixels (460 ppi)	Dual: 12 MP, f/1.6 (wide), 12 MP, f/2.4 (ultrawide)

### Data Preprocessing

The raw images in the SylFishBD dataset were captured in real-world fish markets under highly variable conditions differing lighting, camera angles, resolutions, and file formats. To establish a consistent foundation for annotation and segmentation, a uniform preprocessing pipeline was applied to all 9,075 images. Each image was resized to a fixed resolution of 500  × 500 pixels using bilinear interpolation, with aspect ratio preserved via letterboxing (black padding added symmetrically to shorter dimensions). This approach prevented geometric distortion of fish morphology while maintaining key diagnostic features such as fin structure, scale alignment, body curvature, and eye positioning critical for accurate species identification.

This preprocessing directly enabled:

- **Bounding Box Annotation**: A fixed canvas size reduced bounding box placement variability and improved inter-annotator agreement.
- **Segmentation Mask Generation using SAM**: Consistent input dimensions ensured stable behavior of the Segment Anything Model (SAM) during mask generation and accelerated GPU-based inference.

The complete preprocessing pipeline, implemented in Python using OpenCV and PIL was developed specifically for this work, ensuring complete reproducibility from raw market photographs to the final annotated and masked dataset, though the code is not publicly released.

### Bounding Box Annotation

The SylFishBD dataset was annotated using the LabelImg tool, which supports bounding box annotations for object detection and classification tasks. Each image was carefully labeled to identify the fish species, with bounding boxes drawn around individual fish instances to specify their location and class. Annotations are stored in JSON format, following the structure shown in the provided Table 6, which includes fields such as “label,” “points” (bounding box coordinates), “imagePath,” “imageHeight,” and “imageWidth, mainly saved in COCO format for flexibility. This format ensures compatibility with popular deep learning frameworks like TensorFlow and PyTorch. The annotation process was conducted by trained personnel and cross-verified to ensure high accuracy, making the dataset suitable for developing and evaluating machine learning models for fish species identification in real-world market settings.

**Table 6:** File-format overview with visual example.

### Segmentation Mask Generation using SAM

To elevate the SylFishBD dataset into a multi-task benchmark for advanced computer vision applications-including instance segmentation with high-precision **pixel-level binary segmentation masks** were generated for all 9,075 images using the Segment Anything Model (SAM)^8^. Unlike traditional annotation methods requiring labor-intensive manual polygon labeling, SAM enables automatic mask generation through prompt engineering, demonstrating strong zero-shot transfer capabilities across diverse visual domains^8^. As a foundation model trained on 11 million images, SAM demonstrates remarkable zero-shot instance segmentation capabilities when prompted with coarse annotations such as bounding boxes^9,10^. The segmentation pipeline details in Table 7 leveraged the existing bounding box annotations as prompts to SAM, enabling zero-shot, prompt-guided segmentation of individual fish instances under highly challenging real-world conditions: dense market clutter, partial occlusions, water reflections, vendor interference, overlapping specimens, and variable lighting. This approach ensures tight, object-specific masks that accurately delineate fine anatomical details such as fins, eyes, scales, and body contours. The Post-processing steps included:

- Binarization (threshold = 0)
- Morphological opening with a 3 × 3 kernel to eliminate salt-and-pepper noise
- Selection of the largest connected component to isolate the primary fish region

**Table 7:** Details of the Segment Anything Model (SAM) used for mask generation.

Parameter	Value
Model Variant	ViT-H (632M)
Input Prompt	Bounding box coordinates
Inference Device	NVIDIA T4 GPU
PyTorch Version	2.0.1
SAM Library	segment-anything v1.0
Post-processing	Binarize, 3 × 3 opening, largest component
Output Format	Binary PNG (255 = fish, 0 = background)

**Fig. 2:**
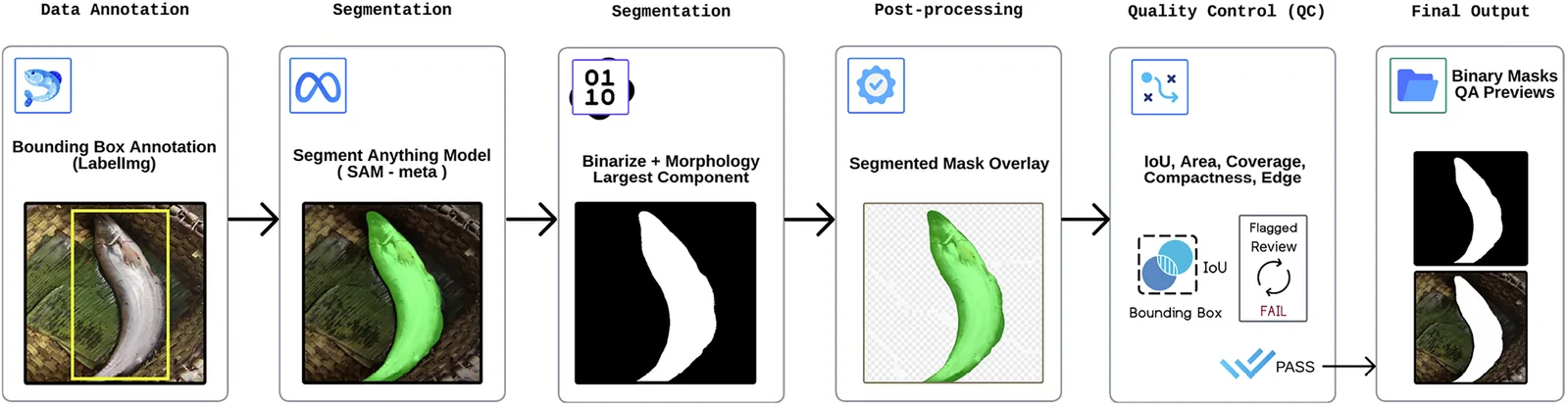
SAM Workflow: Mask Generation from Bounding Box Prompts and Quality Validation.

This Fig. 2 demonstrates the workflow of the Segment Anything Model (SAM), where masks are generated from bounding box (BB) prompts. It also highlights the subsequent quality check stage used to validate and refine the accuracy of the generated segmentation masks.

A rigorous quality control (QC) framework was applied to every generated mask to ensure technical reliability and downstream usability. Each mask was evaluated against six quantitative criteria in Table 8:

**Table 8:** Quality control criteria for segmentation masks.

Criterion	Formula/Condition	Threshold
IoU with BBox	$\,{\rm{IoU}}\,=\frac{| M\cap B| }{| M\cup B| }$	≥0.75
Mask-to-BBox Area	$\frac{| M| }{| B| }$	0.4–0.95
Image Coverage	$\frac{| M| }{W\times H}$	≤0.70
Compactness	$4\pi \cdot \frac{| M| }{{P}^{2}}$	≥0.2
Continuity	Connected components	≤2
Edge Contact	% of mask boundary on image edge	≤70%

Masks failing any critical threshold (IoU, area ratio, coverage, edge contact) were automatically discarded and logged for review. This resulted in a near-perfect success rate, with all 9,075 masks passing QC and demonstrating excellent boundary adherence across species from elongated forms (boal, ilish) to compact ones (koi, pabda).

**Fig. 3:**
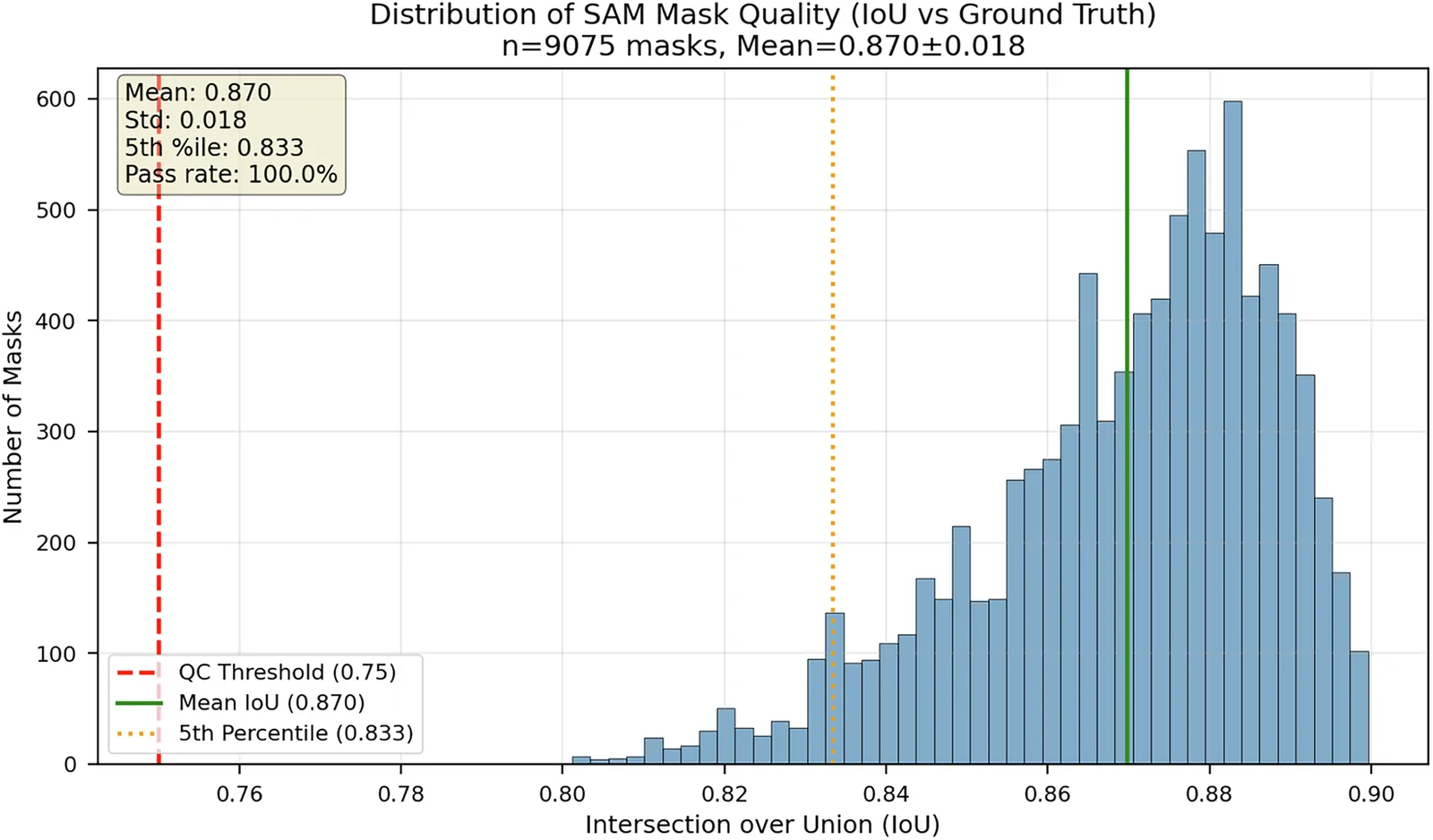
Distribution of SAM mask quality (IoU) across 9,075 masks.

The IoU distribution for all 9,075 masks (Fig. 3) shows a mean of 0.870 (±0.018). The 5th percentile is 0.833, and the minimum IoU of 0.788 still exceeds the 0.75 threshold by +0.038. The distribution is left-skewed and centered around 0.87–0.88, with 100% of masks passing QC. No borderline cases (IoU < 0.78) were observed. The minimum IoU of 0.788 ensures all masks surpass the 0.75 threshold with a 5.1% margin, indicating consistently high-quality segmentation across all 9,075 samples, likely due to clear foreground-background contrast in aquaculture images.

The final output is structured for immediate integration into modern frameworks:

- Binary PNG masks stored under masks/{class}/{class}_mask_{id}.png
- Pixel values: **255 = fish**, 0 = background

### Complete Dataset Segmentation and Annotation

Table 9 presents sample segmentation results for all nine fish classes. For each class, the table shows the original image with bounding boxes, the corresponding segmentation mask generated by SAM, and the overlay of the mask on the fish to illustrate the accuracy and quality of segmentation.

**Table 9:** Sample Segmentation Results for All Nine Fish Classes.

### Experimental Design

The SylFishBD dataset was specifically curated to enable automated fish species identification in real-world local fish markets of Bangladesh, addressing critical challenges such as species mislabeling, price manipulation, and lack of consumer transparency. A total of 9,075 high-quality images containing 9,075 annotated fish instances across nine prevalent freshwater species were collected from five distinct fish markets in Sylhet, Bangladesh (coordinates: 24.8911^°^N, 91.8700^°^E) over a 6–7 month period to capture seasonal variations in fish morphology, coloration, and market presentation (e.g., fresh vs. iced, live vs. processed).

**Fig. 4:**
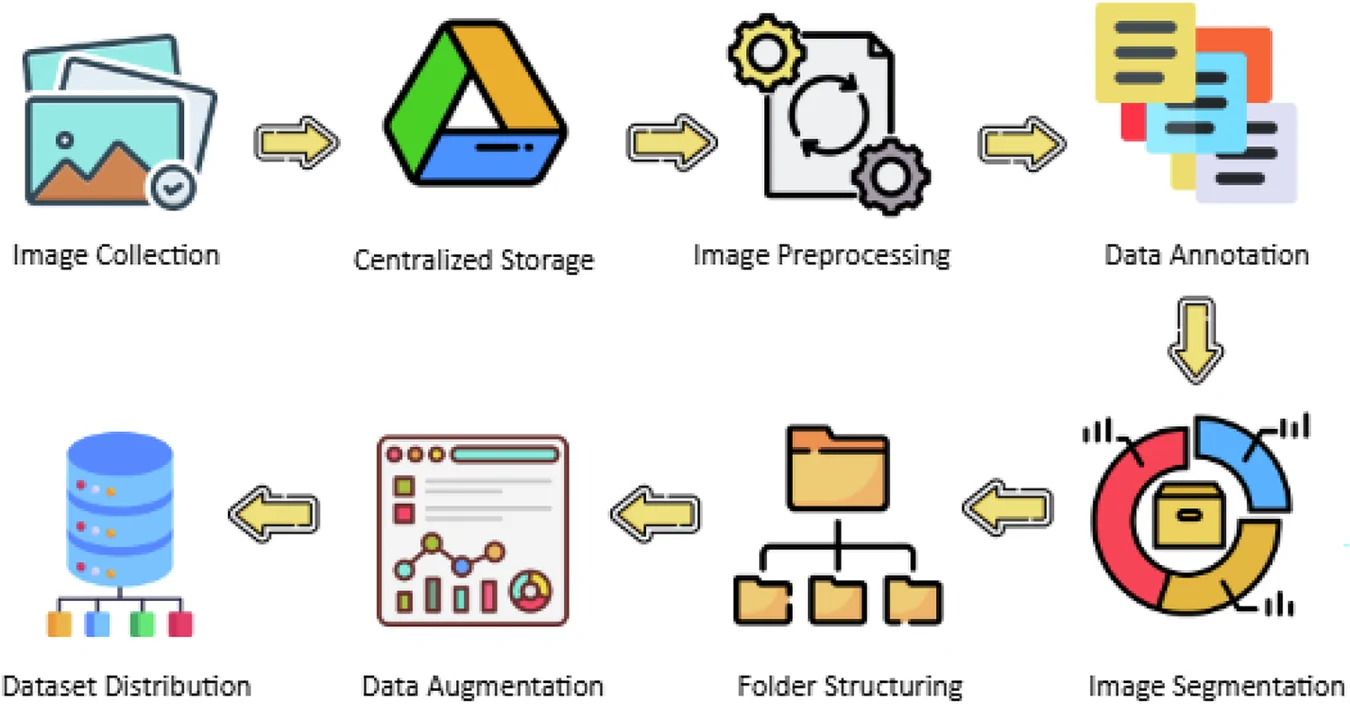
Complete methodology workflow for the SylFishBD dataset.

This strict one-fish-per-image policy, combined with comprehensive annotation and mask generation, ensures that SylFishBD is a clean, unambiguous, and highly challenging benchmark for real-world fish species recognition. The dataset faithfully reflects the true complexity of operational fish markets, enabling robust training and evaluation of vision models for practical deployment, such as mobile apps for consumer education, automated pricing systems, or regulatory market monitoring tools. The complete experimental methodology is visually summarized in Fig. 4.

An initial pool of approximately 10,000 images was gathered. After rigorous quality filtering, 925 images were excluded due to:

- Severe motion blur or focus issues
- Low resolution (<720p effective detail)
- Extreme overexposure or underexposure
- Heavy compression artifacts or corrupted files

Of the 925 excluded images 9.3% rejection rate, most were due to motion blur 42% (389 images), followed by low resolution 23% (213 images), exposure issues 19% (176 images), and compression artifacts 16%(147 images), indicating motion blur as the primary challenge. This resulted in a final curated set of 9,075 images, each containing exactly one clearly visible and fully annotated fish instance. Every image includes:

- A tight bounding box around the fish
- A binary segmentation mask generated using the Segment Anything Model (SAM)

#### Inclusion and Exclusion Criteria

- **Inclusion**:
- Single, well-centered fish per image
- Captured under authentic market lighting and environmental conditions
- Diverse fish orientations, sizes, and freshness states
- Real-world clutter and background variability to promote model robustness
- **Exclusion**:
- Multiple fish per image (to avoid annotation ambiguity)
- Blurry or out-of-focus subjects
- Low-resolution or heavily compressed files
- Overexposed, underexposed, or artificially lit studio shots

The current dataset employs a strict one-fish-per-image policy to eliminate annotation ambiguity and ensure high-quality, unambiguous ground truth labels. While this design choice sacrifices some ecological realism, as fish are frequently displayed in groups and stacked arrangements in real markets, it provides a clean benchmark for initial model development. We recognize that multi-fish scenarios represent an important extension for practical deployment, and future dataset versions may incorporate multi-instance images with corresponding instance-level annotations.

## Data Records

### Dataset Access

The SylFishBD dataset is publicly available and hosted on Kaggle^11^, a widely used platform for open data sharing and machine learning competitions, ensuring long-term accessibility, version control, and seamless integration with cloud-based training environments (see Table 10).

**Table 10:** Dataset access and download information.

**Repository**	https://kaggle.com/shakibabsar42/SylFishBD (*Kaggle*)
**Total Size**	approximately 336.19 MB (zip file)
**Download Format**	Single .zip archive

### File Naming Convention

The dataset employs a consistent naming scheme across all file types to ensure easy file matching and retrieval (see Table 11).

**Table 11:** File Naming Patterns.

Type	Pattern	Example	Description
Image	{class}_{number}.jpg	boal_001.jpg	Original fish image (500 × 500 px)
Annotation	{class}_bb_{number}.json	boal_bb_001.json	Bounding box coordinates in JSON format
Mask	{class}_mask_{number}.png	boal_mask_001.png	Binary segmentation mask (white = fish, black = background)

### Folder Structure

**Fig. 5:**
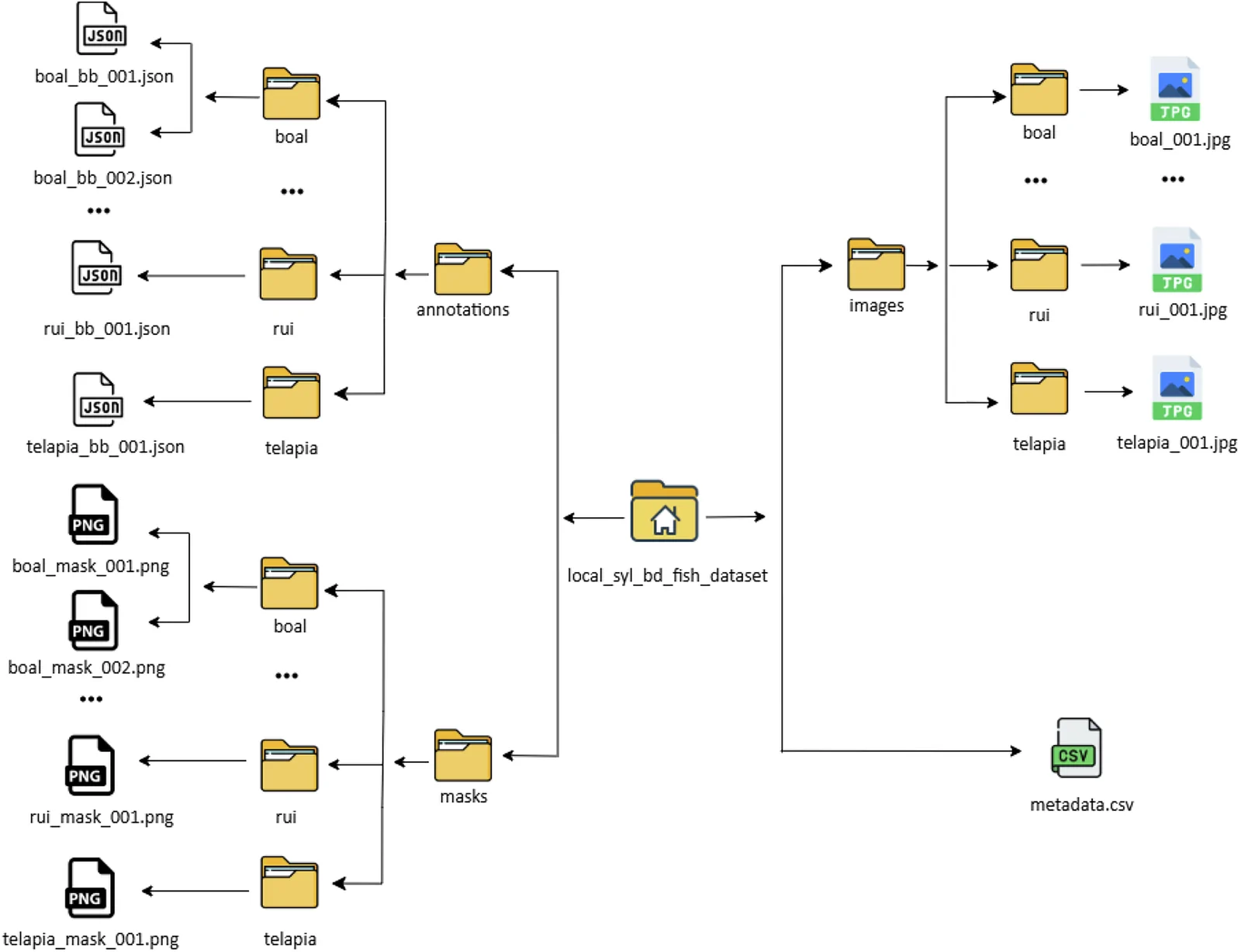
Folder structure.

Figure 5 depicts the dataset’s structured hierarchy, featuring three primary directories for images, annotations, and masks, complemented by metadata files at the root level, ensuring organized and accessible data management.

#### File Relationships

Each fish sample maintains a three-way relationship between its image, annotation, and mask files. All three files share the same sequential index number for easy cross-referencing. For example, for the first boal fish:

- boal_001.jpg  ↔ Original image (500 × 500 pixels)
- boal_bb_001.json  ↔ Bounding box coordinates
- boal_mask_001.png  ↔ Binary segmentation mask (0/255)

#### Root Files

The dataset includes two essential root-level metadata files that facilitate rapid indexing, class reference, and integration with machine learning pipelines.

- classes.txt: A plain-text file listing the nine fish species in alphabetical order, one per line. This file serves as the canonical source for class labels and is used by training scripts to map string names to integer IDs.
- metadata.csv: A comprehensive, machine-readable index containing one row per image (9,075 total). Each entry provides full file linkage and basic image properties, enabling efficient data loading, filtering, and validation without traversing the directory tree.

These files enable programmatic dataset inspection, stratified splitting, and seamless integration with data loaders in PyTorch, TensorFlow, and MMDetection. The metadata.csv file whose schema is given in Table 12 is particularly valuable for reproducibility, as it decouples data organization from file system assumptions.

**Table 12:** Schema of metadata.csv.

Column	Description
image_id	Unique identifier without extension (e.g., boal_001)
class_name	Fish species (matches classes.txt)
image_path	Relative path to JPEG (e.g., images/boal/boal_001.jpg)
annotation_path	Relative path to bounding box JSON (e.g., annotations/boal/boal_bb_001.json)
mask_path	Relative path to binary mask PNG (e.g., masks/boal/boal_mask_001.png)
width	Image width in pixels (500)
height	Image height in pixels (500)
file_size_kb	Size of the image file in kilobytes (rounded to two decimals)

## Data Overview

### Distribution Analysis

**Fig. 6:**
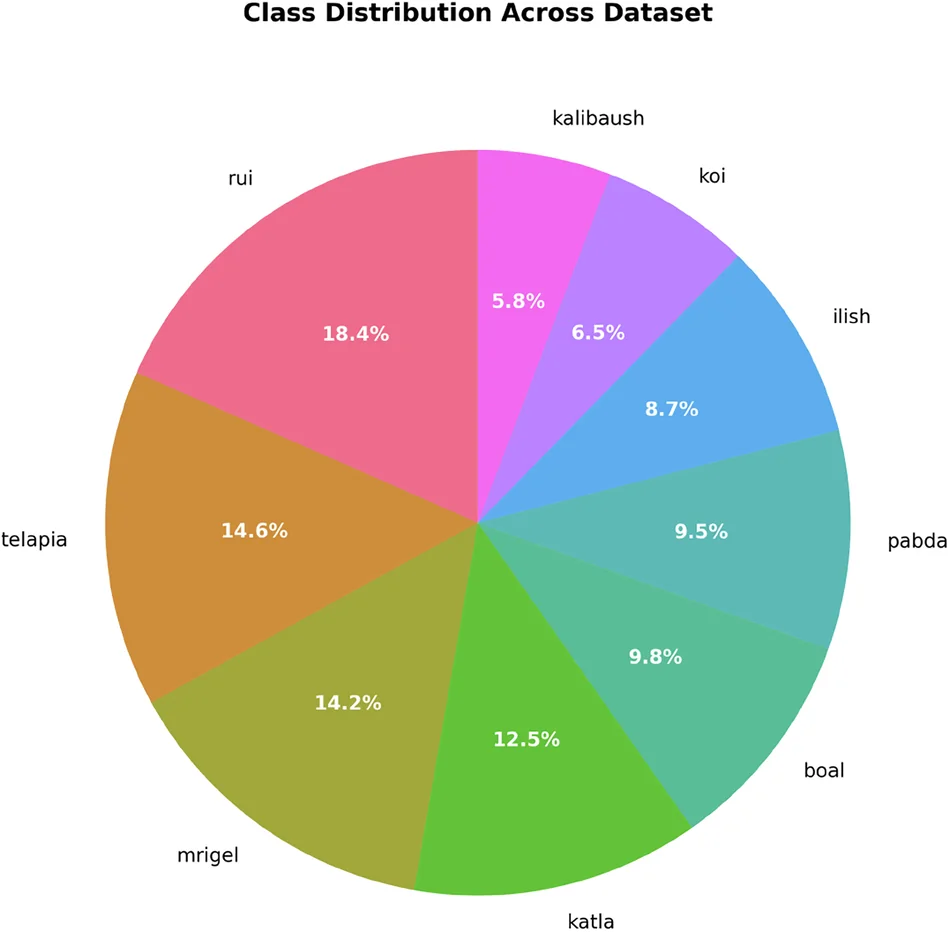
Species distribution across the SylFishBD dataset.

The SylFishBD dataset comprises a total of 9075 images containing annotated fish instances and their corresponding masks across nine freshwater species commonly traded in Bangladeshi markets. Unlike typical machine learning datasets, SylFishBD is released as a complete, unified benchmark without predefined training, validation, or test splits (see Fig. 6). This design choice enables maximum flexibility for researchers to define custom splits stratified by species, market location, lighting conditions, or capture date tailored to specific experimental needs such as cross-validation, domain adaptation, or few-shot learning Table 13.

**Table 13:** Data access and archival information.

Platform	Details
**Kaggle (Primary)**	https://www.kaggle.com/datasets/shakibabsar42/sylfishbd
	***DOI***: https://doi.org/10.34740/kaggle/ds/8761062
	***Version***: 1.0 (initial release)
	***Size***: ~336.19 MB
	***Files***: 9,075 images, 9,075 JSON annotations, 9,075 PNG masks, metadata, and documentation

The distribution of specimens per species is intentionally balanced to reflect real-world market prevalence while ensuring sufficient representation of both dominant and less common species. Table 14 provides a detailed breakdown of each class, including local (Bangla), English, and scientific names, along with the total number of annotated instances.

**Table 14:** Overview of the SylFishBD dataset: species distribution and specimen counts.

Sl.	Local Name	English Name	Scientific Name	Number of Specimens
1	Rui	Rohu	*Labeo rohita*	1,670
2	Telapia	Tilapia	*Oreochromis mossambicus* / *Oreochromis niloticus*	1,326
3	Mrigel	Mrigal Carp	*Cirrhinus cirrhosus*	1,293
4	Katla	Catla	*Catla catla*	1,133
5	Boal	Wallago	*Wallago attu*	885
6	Pabda	Pabda Catfish	*Ompok pabda*	862
7	Ilish	Hilsa	*Tenualosa ilisha*	789
8	Koi	Climbing Perch	*Anabas testudineus*	592
9	Kalibaush	Orange-fin Labeo	*Labeo calbasu*	525
**Total**				**9,075**

The dataset exhibits a natural long-tail distribution: high-abundance species like Rui (18.4%) and Telapia (14.6%) dominate, consistent with aquaculture and market supply trends, while smaller species like Koi and Kalibaush remain sufficiently represented for robust modeling. To address class imbalance (3.2:1), we recommend using stratified splits, class-weighted loss functions, and balanced sampling (over/under-sampling), along with reporting per-class metrics to ensure fair evaluation across all classes. The Telapia class groups Oreochromis mossambicus and Oreochromis niloticus, which are difficult to distinguish visually and are treated as a single category in local markets; this may limit fine-grained classification. All images include corresponding bounding box annotations and high-quality binary segmentation masks generated via the Segment Anything Model (SAM), making SylFishBD a comprehensive resource for classification, object detection, and instance segmentation tasks in real-world aquatic environments.

## Technical Validation

The dataset underwent rigorous quality control procedures to ensure technical integrity, reliability, and usability for machine learning applications. Multiple validation steps were implemented to verify data consistency, annotation accuracy, and mask quality.

### Image Characteristics

#### Dimension Consistency

**Fig. 7:**
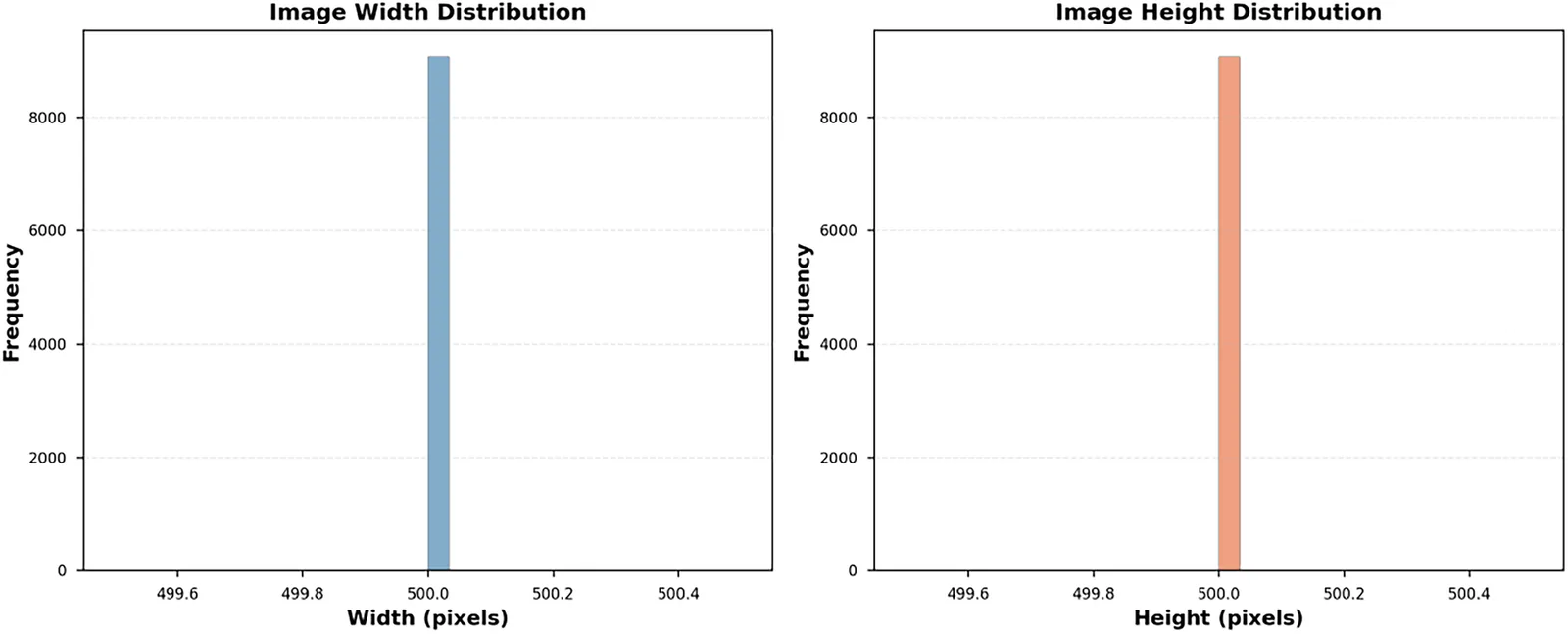
Distribution of image widths and heights across the dataset, confirming uniform 500 × 500 pixel resolution.

All images in the dataset maintain a consistent resolution of 500 × 500 pixels, as verified through automated dimension checking. Figure 7 shows the distribution of image dimensions across the dataset.

#### File Size Analysis

**Fig. 8:**
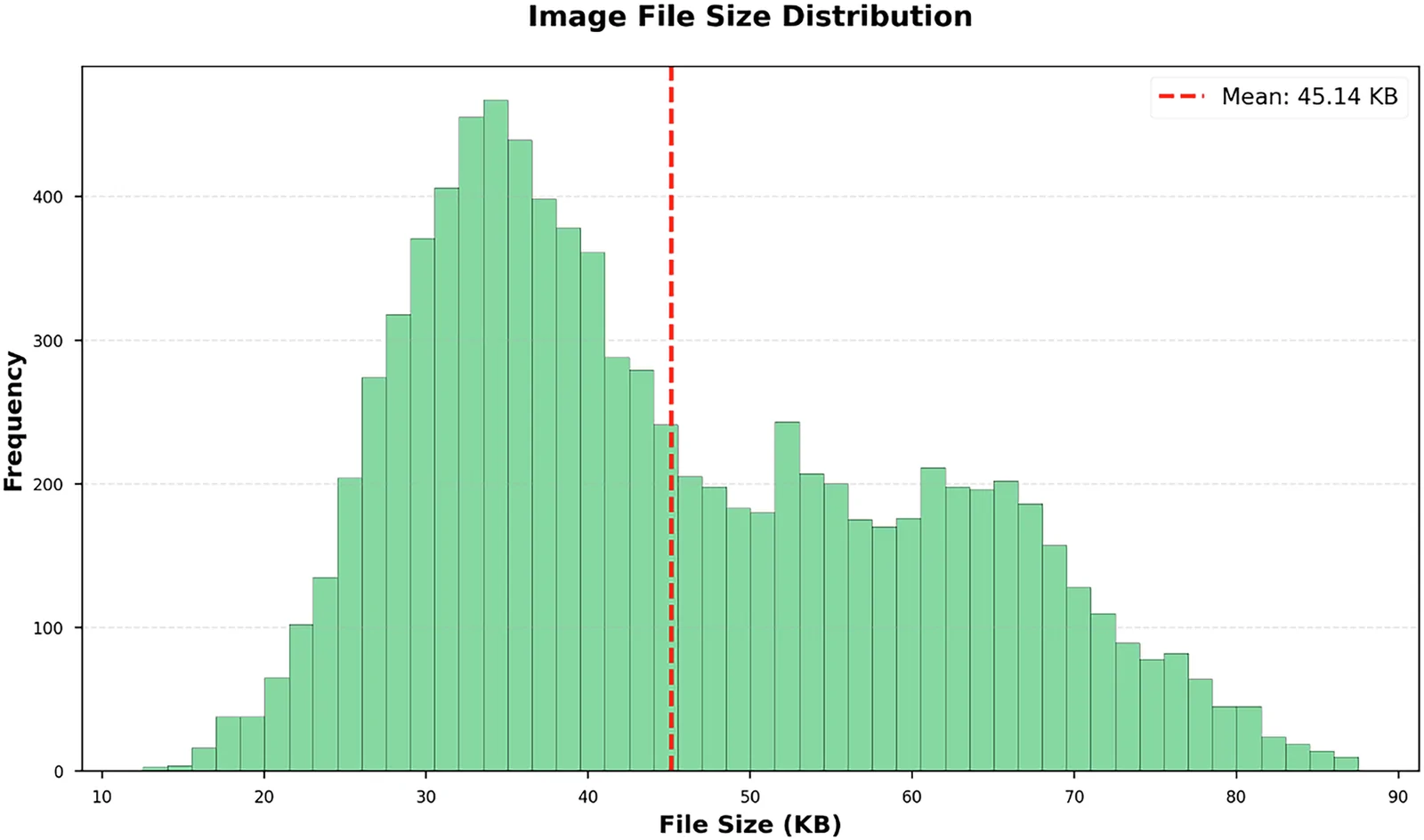
Distribution of image file sizes in kilobytes, showing consistent JPEG compression across the dataset.

Image file sizes range from 15 to 80 KB with a mean of approximately 45 KB. The distribution shown in Fig. 8 indicates consistent compression quality across the dataset.

### Baseline Model Performance

#### Object detection

Evaluation of object detection performance on the SylFishBD dataset revealed exceptional accuracy across both YOLO architectures (Table 15). The YOLOv5s model achieved a precision of 0.990, recall of 0.993, mAP@0.5 of 0.994, and mAP@0.5:0.95 of 0.883. The YOLOv8s architecture demonstrated comparable performance with a mAP@0.5 of 0.994, and mAP@0.5:0.95 of 0.887. Notably, YOLOv8s exhibited marginally superior localization accuracy (higher mAP@0.5:0.95) despite requiring 22% more parameters (11.13M vs. 9.13M), suggesting improved bounding box regression capabilities. Both models maintained consistently high F1-scores (>0.99) across all nine fish species, indicating robust detection performance with minimal false positives or missed detections.

**Table 15:** Object detection and segmentation benchmark results on the SylFishBD dataset.

Task	Model	Params (M)	mAP@0.5	mAP@0.5:0.95
Object Detection	YOLOv5s	9.13	**0.994**	0.883
	YOLOv8s	11.13	**0.994**	**0.887**
Semantic Segmentation	U-Net (ResNet34)	24.4	0.9301 (mIoU)	0.9428 (Dice)
	DeepLabV3+ (ResNet50)	42.0	**0.9375** (mIoU)	**0.9510** (Dice)

#### Semantic segmentation

Segmentation performance was evaluated using U-Net and DeepLabV3+ architectures, both demonstrating high-fidelity pixel-level classification (Table 15). DeepLabV3+ achieved superior performance with a mean IoU of 0.9375 and Dice coefficient of 0.9510, likely benefiting from its atrous spatial pyramid pooling and encoder-decoder structure. U-Net achieved competitive results with a mean IoU of 0.9301 and Dice coefficient of 0.9428. The sub-1% performance gap between architectures suggests that the high-contrast aquaculture imaging conditions facilitate robust segmentation regardless of network topology. Both models achieved per-class IoU scores exceeding 0.92 for all species, with *ilish* and *boal* exhibiting marginally lower scores (IoU  ~ 0.93) potentially attributable to their elongated body morphologies.

## Usage Notes

The SylFishBD dataset is designed for immediate use in machine learning and computer vision research. The following technical notes facilitate efficient access, processing, and integration:

- **Data Loading**: Use metadata.csv as the primary index for batch loading. Each row provides full relative paths to the image (image_path), bounding box annotation (annotation_path), and segmentation mask (mask_path). This enables direct construction of data loaders in PyTorch, TensorFlow, or MMDetection without directory traversal.
- **File Formats and Compatibility**:
- Images are 8-bit RGB JPEGs (500  × 500 pixels), compatible with all standard image loaders.
- Bounding box annotations are stored in individual JSON files following a COCO-style schema, allowing seamless import into Detectron2, MMDet, or YOLO via conversion scripts.
- Binary segmentation masks are PNGs with pixel values 255 (fish) and 0 (background), directly usable in instance segmentation frameworks such as Mask R-CNN or YOLACT.

These notes ensure reproducible and efficient use of SylFishBD across diverse research and deployment scenarios.

## Data Availability

The dataset is available at Kaggle^11^ to ensure long-term discoverability, reproducibility, and compliance with open science standards (Table 13): The dataset is released under the **Creative Commons CC0 Public Domain Dedication (CC0 1.0). Although attribution is not legally required, users are encouraged to cite the associated publication when reusing the dataset. ** All files are provided in standard, non-proprietary formats: • **Images**: 8-bit RGB JPEG, 500  × 500 pixels • **Annotations**: Individual JSON files (COCO-compatible structure) • **Masks**: Binary PNG (255 = foreground, 0 = background) • **Metadata**: metadata.csv and classes.txt. The dataset will not be modified post-publication; any future extensions will be released under a new version.

## Code availability

All code used for dataset preparation, preprocessing, and figure generation for the dataset is openly available at: https://github.com/sabsar42/sylfishbd-dataset-codes. This includes scripts used to organize the dataset, generate segmentation masks, structure file naming, and prepare the final release. Metadata is also stored at these links.
